# Long noncoding RNA 00976 promotes pancreatic cancer progression through OTUD7B by sponging miR-137 involving EGFR/MAPK pathway

**DOI:** 10.1186/s13046-019-1388-4

**Published:** 2019-11-20

**Authors:** Shan Lei, Zhiwei He, Tengxiang Chen, Xingjun Guo, Zhirui Zeng, Yiyi Shen, Jianxin Jiang

**Affiliations:** 1 Department of Hepatobiliary Surgery, Renmin Hospital of Wuhan University, 99 Ziyang Road, Wuhan City, Hubei Province 430060 People’s Republic of China; 2 Key Laboratory of Tissue Engineering and Stem Cell of Guizhou Province, Department of Physiology, School of Basic Medicine, Guizhou Medical University, Guiyang, 550009 Guizhou China; 3 Department of Hepatic-Biliary-Pancreatic Surgery, The Affiliated Hospital of Guizhou Medical University, Guiyang, China; 4 Department of Biliary-Pancreatic Surgery, Affiliated Tongji Hospital, Tongji Medical College, Huazhong University of Science and Technology, Wuhan, 430060 China; 5 Hubei Key Laboratory of Digestive System Disease of Wuhan University, Wuhan, 430060 China

**Keywords:** Linc00976, Pancreatic cancer, miR-137, OTUD7B, EGFR

## Abstract

**Background:**

Accumulation evidence indicates the vital role of long non-coding RNAs (lncRNAs) in tumorigenesis and the progression of malignant tumors, including pancreatic cancer (PC). However, the role and the molecular mechanism of long non-coding RNA 00976 is unclear in pancreatic cancer.

**Methods:**

In situ hybridization (ISH) and qRT-PCR was performed to investigate the association between linc00976 expression and the clinicopathological characteristics and prognosis of patients with PC. Subsequently, linc00976 over-expression vector and shRNAs were transfected into PC cells to up-regulate or down-regulate linc00976 expression. Loss- and gain-of function assays were performed to investigate the role of linc00976 in proliferation and metastasis in vitro and vivo. ITRAQ, bioinformatic analysis and rescue assay were used to illustrate the ceRNA mechanism network of linc00976/miR-137/OTUD7B and its downstream EGFR/MAPK signaling pathway.

**Results:**

linc00976 expression was overexpressed in PC tissues and cell lines and was positively associated with poorer survival in patients with PC. Function studies revealed that linc00976 knockdown significantly suppressed cell proliferation, migration and invasion in vivo and in vitro, whereas its overexpression reversed these effects. Based on Itraq results and online database prediction, Ovarian tumor proteases OTUD7B was found as a downstream gene of linc00976, which deubiquitinated EGFR mediates MAPK signaling activation. Furthermore, Bioinformatics analysis and luciferase assays and rescue experiments revealed that linc00976/miR137/OTUD7B established the ceRNA network modulating PC cell proliferation and tumor growth.

**Conclusion:**

The present study demonstrates that linc00976 enhances the proliferation and invasion ability of PC cells by upregulating OTUD7B expression, which was a target of miR-137. Ultimately, OTUD7B mediates EGFR and MAPK signaling pathway, suggesting that linc00976/miR-137/OTUD7B/EGFR axis may act as a potential biomarker and therapeutic target for PC.

**Electronic supplementary material:**

The online version of this article (10.1186/s13046-019-1388-4) contains supplementary material, which is available to authorized users.

## Background

Pancreatic cancer is currently one of the most severe gastrointestinal malignancies. The symptoms are atypical, advanced disease progression is rapid, and there are no sensitive early diagnostic indicators or effective treatment initiation points in the clinic [1]. The development of pancreatic cancer is a biological process involving multiple genes and multiple steps [2]. Although studies have made great progress on many levels and in many fields, including genes, proteins and cells, much of the malignant biological mechanism of pancreatic cancer remains to be elucidated [3, 4]. Therefore, further study about the progress of pancreatic cancer, especially the search of effect therapy target, is of great significance for improving the curative effect and prognosis of pancreatic cancer. Epigenetic regulation, including noncoding RNAs (which can be divided into long and short noncoding RNAs, according to their length), plays an important role in the complex regulatory network in the process of stem cell differentiation and tumor development, providing new ideas and research directions for the pathogenesis and treatment of tumors [5]. Studies have shown that there are approximately 10,000–20,000 human lncRNAs that contain poorly conserved sequence, are unstable [6], and occur at low copy numbers. However, they can regulate gene expression at multiple levels of epigenetic regulation, by affecting DNA methylation, histone modification, random chromosome inactivation, coding and other noncoding RNAs, and small peptides, without changing the nuclear acid sequence [7, 8]. Long noncoding RNAs (lncRNAs) and microRNAs (miRNAs) constitute the majority of regulatory noncoding RNAs [9, 10]. miRNAs are function as vital regulator in multiple Physiological and pathological processes and play a critical role in mRNA stability and translation by post-transcriptionally regulation at 3′ untranslated regions (3′-UTRs) of mRNAs [11].

Recently, there has gradually been discovered in the study of deubiquitination enzymes in cancer and other disease. Deubiquitination enzyme OTUD7B belongs to the ovarian tumor family of deubiquitination enzymes, and is similar to that of the cysteine proteases, but it has different functions [12–15]. The mechanism of the association between OTUD7B and the occurrence and development of tumor cells is unclear, and the specific function of its action on cells has not been fully clarified. Studies have shown that OTUD7B can act on EGFR to affect the activation of the EGFR signaling pathway [16, 17].

In this research, Linc00976 was significantly overexpressed in PC and correlative with clinical pathology features in PC patients. Function assays eluted that Linc00976 promoted proliferation and metastasis of PC cells both in vitro and in vivo via up-regulating its host gene OTUD7B involving EGFR deubiquitination and downstream pathway MAPK activation. Further mechanistic studies indicated that Linc00976 exerts oncogene function by competitively interacting with miR-137 and acting as a microRNA sponge, thus inhibiting miR-137 and upregulating OTUD7B expression.

## Materials and methods

### Cell culture

The cell lines we used in the study were purchased from American Type Culture Collection (ATCC). CFPAC-1, MIA-PaCa-2 and PANC-1 cells were cultured in high-glucose DMEM (Gibco, NY, USA), while BxPC-3, CFPAC-1, ASPC-1, Panc03.27, and Capan-2 cells were cultured in RPMI-1640 (Gibco, NY, USA). The cells used in the research were MIA-PaCa-2 and PANC-1. All the cell lines were cultured at 37 °C in a humidified atmosphere containing 5% CO_2_.

### qRT-PCR, western blotting analysis

qRT-PCR and Western blotting analysis was performed as described previously [19]. The reagents of qRT-PCR used in the experiments were all purchased from Takara, Japan. The Primer sequences used for performing qRT-PCR assay are shown as Table 1.The primary and second antibodies used for western blotting of CyclinD1, CDK2, MMP2, MMP9, EGFR, SAPK/JUK, p- SAPK/JUK, P38, p-P38, ERK, p-ERK, p-MEK, OTUD7B and GAPDH, were all purchased from CST company, the dilution ratio is according to the instructions.

**Table 1:** The characteristics of the primers used for real-time PCR and plasmid sequence

Gene	Sequence (5′ - > 3′)	
Linc00976	Forward Primer	CAGCAGTGTGAAGCAATG
	Reverse Primer	ACAAGTAGAGTCTATGGTTCC
OTUD7B	Forward Primer	GTCAGATTTTGTCCGTTCCACA
	Reverse Primer	CATGGACTTGACGTAGCTGTT
CyclinD1	Forward Primer	GCTGCGAAGTGGAAACCATC
	Reverse Primer	CCTCCTTCTGCACACATTTGAA
CDK2	Forward Primer	CCAGGAGTTACTTCTATGCCTGA
	Reverse Primer	TTCATCCAGGGGAGGTACAAC
MMP2	Forward Primer	TACAGGATCATTGGCTACACACC
	Reverse Primer	GGTCACATCGCTCCAGACT
MMP9	Forward Primer	TGTACCGCTATGGTTACACTCG
	Reverse Primer	GGCAGGGACAGTTGCTTCT
Control	UUAUUGCUUAAGAAUACGCGUAG	
	UCACAACCUCCUAGAAAGAGUAGA	
miR-137 inhibitor	CTACGCGTATTCTTAAGCAATAA	
miR-137 mimic	UUAUUGCUUAAGAAUACGCGUAG	
Si-Linc00976	5′-GTCAATTGCTCAAACCAAA-3’	
Si-OTUD7B	GCAAAGAUGAUAGUGACAATT	
	UUGUCACUAUCAUCUUUGCTT	

### RNA fluorescence in situ hybridization analysis (FISH)

The different groups cells were seeded in the confocal dish at a density of 2 × 10^5^.The paraformaldehyde (4%) was used to fixed the cell at 37 °C for 30 min. According to the manual, the probe was added into the dish in the dark at indicated temperature and time.

After twice washing with PBST, the cells were stained with DAPI and incubated for 5 min at 37 °C. After twice washing with PBS containing Tween-20 (PBST), the cells were blocked using glycerin. Images were captured using a microscopy (ZEISS) at 400× magnification. The regents used in the FISH assay was purchased from Ribobio (Guangzhou, China).

### Cell viability analysis

For the Cell Counting Kit-8(CCK-8) assay, according to the instruction (Dojindo, Japan), the different group cells were plated in 96-well plates with a density of 4000 cells. After culturing 0, 24, 48, 72 and 96 h, 200 μl medium contained of CCK-8 regent was fixed and then added into the wells. After incubating for the indicated time, the absorbance value was detected at 450 nm with a microplate reader.

### Colony formation assay

Pancreatic cancer cells were inoculated in culture dishes at 1000 cells/plate. They were placed at 37 °C and 5% CO_2_ for 10 days, and then the medium was discarded. The paraformaldehyde (4%) was used to fixed the cells 15 min, and then crystal violet was used to stain the cells 30 min. The clones were counted after imaging, and statistical analysis was performed on the results of the count.

### Cell cycle assay

According to the manufacturer’s protocol of BD (Becton, Dickinson and Company, USA) cell cycle regent. Pancreatic cancer cells were collected after centrifugation in the PBS. After fixing in 75% ethanol for 24 h, the mixture was centrifuged, and the supernatant was discarded. After washing away the residual ethanol with PBS, the cells were stained with 200 μl PBS contained PI (propidium iodide) 10 μ incubated for 5 min at 37 °C. At last, the cell suspension was detected and analyzed in the flow cytometer.

### EdU assay

The different groups cells were seeded in the confocal dish at a density of 5 × 10^5^.The paraformaldehyde (4%) was used to fixed the cell 10 min. After washing with PBS, the triton (1%) was used to transparent the cell for 5 min. According to the manual, the cell was incubated with dyeing agent for 30 min in the dark and stained with 1 × Hochest33342 and incubated for 5 min at 37 °C. After twice washing with PBS containing Tween-20 (PBST) the images were captured using a microscopy (ZEISS) at 400× magnification. The regents used in the EDU assay was purchased from Ribobio (Guangzhou, China).

### Wound healing assay

The different groups cells were plated in six-well plates and allowed to grow to confluence. The cell wound was scratched a 200 μl pipette tip. The indicated cells were deprived of serum for 48 h, treated with mitomycin-C. Photos of migration were captured at 0 and 48 h after scraping.

### Transwell assays

The indicated cells were deprived of serum were seeded in the upper Transwell chamber, and the complete medium contained 10% FBS was added into the bottom chamber. After cultured in the normal condition for 24 h, the cell in the upper chamber would wipe out by cotton swab, and the migrated cells were fixed with paraformaldehyde and stained with crystal violet. The transwell invasion experiment required matrigel to be coated in the upper chamber before cell seeding.

### In vivo assay

For the proliferation assays, PANC-1 cells of linc00976 knockdown, linc00976 overexpressed and negative control were subcutaneously injected into BALB/c nude mice. The mice were weighed every week and euthanized in 7 weeks after injection. The tumors were dissected and weighed (4–6 weeks old, female, *n* = 6 per group).

For the metastasis assays, spleen capsule injected liver metastasis model was used to illustrate the different groups PC cells metastasis ability. A total of 1× 10^6^ cells in 150 μL PBS were injected into the spleen. of nude mice. The mice were weighed every week and euthanized 12 weeks after injected. The liver tissues were harbested and photo. IHC was used to detected the metastasis loci in the liver.

All conditions and procedures for the animal experiments were approved by the Animal Care Committee of WuHan University.

### ISH (in situ hybridization)

Experiments were performed with the RNA ISH Kit (BersinBi, Beijing, China) according to the manufacturer’s instructions. Briefly, PC cells were fixed in 4% paraformaldehyde for 20 min, washed with distilled water, treated with pepsin (1% in 10 mM HCl), and incubated with 20 nM ISH probe in hybridization buffer (100 mg/mL dextran sulfate, 10% formamide in 2X SSC) at 90 °C for 3 min. Hybridization was performed at 37 °C for 18 h, followed by a wash step and an incubation with digoxin antibodies for 1 h. Finally, DAB was applied to the samples to detect the signals. The ISH images were captured using an Aperio ImageScope system. Each sample was examined by two pathology specialists who were blinded to the diagnoses and outcomes. Staining intensities and percentages of positive cells were recorded. The relative expression of each sample was calculated as the product of the expression intensity and percentage of positivity.

### iTRAQ (isobaric tags for relative and absolute quantitation)

Lysis buffer containing 8 M carbamide, 30 mM HEPES, 1 mM PMSF, 2 mM EDTA, and 10 mM DTTwas added to cells and centrifuged at 20,000×g and 4 °C for 30 min to collect the supernatant. Protein concentrations were determined using the Bradford method. For digestion, each sample was reduced with 10 mM dithiothreitol for 60 min at 56 °C and alkylated using 55 mM iodoacetamide for 60 min at room temperature in the dark. The proteins were digested with 1 μg/μl trypsin at a weight ratio of 1:30 (trypsin:protein) overnight at 37 °C. Tryptic peptides were lyophilized and resuspended in 0.5 M Triethylammonium bicarbonate. Following trypsin digestion, each iTRAQ reagent was dissolved in isopropanol and added to the appropriate peptide mixture. A total of 3 biological replicates of the knockdown of Linc00976 group were labelled with iTRAQ tags 113, 114 and 115, respectively. Similarly, 3 biological replicates of the negative control group were labelled with iTRAQ tags 118, 117 and 116, respectively. The labelled peptide mixtures were incubated at room temperature for 2 h and obtained by vacuum-drying. Then, peptides were desalted using a Strata X C18 SPE column (Phenomenex, Torrance, CA, USA), and analyzed using a mass spectrometer (TripleTOF 6600; SCIEX, Framingham, MA, USA). The instrument parameters were as follows: Mass range, 350–2000 m/z for time-of-flight mass spectrometry (TOF MS) and 100–1500 m/z for TOF MS/MS; dynamic exclusion, 12.0 s. Mass spectra raw data were analyzed with ProteinPilotä software (version 5.0; SCIEX). Peptides were identified using a false discovery rate of < 1%. Proteins were considered differentially expressed if they differed in at least 2 of the 3 biological replicates. The criteria of *P* < 0.05 and fold change (ET/LPS) > 1.2 were selected to identify up- and down-regulated proteins.

### Reagents and transfections

We purchased siRNAs from RiboBio (Guangzhou, China). We purchased human Linc00976 over-expressing, knocking-down and negative-control lentiviruses from Genechem (Shanghai, China). Linc00976 mutant plasmids were purchased from Genechem (Shanghai, China). All transfections were carried out according to manufacturers’ instructions.

### Luciferase reporter assay

PC cells were plated into the 6-well plates at dentist of 5 × 10^5^.Then the corresponding psiCHECK™-2 vector and miR-137 mimics were co-transfected into the indicated well. According to the manufacturer’s protocol (Ribobio, Guangzhou), the lysate was used to measure the firefly and Renilla luciferase activities. Then, the relative luciferase activity was normalized to the firefly luciferase internal control.

### Immunoprecipitation

PC cells were lysed in lysis buffer to be extracted, and then the BCA method was used to quantity the cell lysates concentration. Cell lysates were incubated in primary antibody at 4 °C overnight and Protein A Sepharose beads was added into lysates for 4 h. The beads were boiled in Loading buffer (2×) after pre-cooled PBS washed. The protein was detected with western blot or Mass spectrometric analysis.

### Statistical analyses

Results were eluted as mean ± SD. Statistical analysis was performed using GraphPad Prism 7.0 (San Diego, CA, USA). Chi-square test was used to analyze the correlation between linc00976 expression levels and clinicopathological features in PC. Kaplan–Meier curve plots method was used to analyze overall free survival rate. Student’s t-test was used for statistical comparison between different groups. *P < 0.05, **P < 0.05.

## Results

### The expression of Linc00976 and its correlation with the clinical parameters in patients with PC

**Fig. 1:**
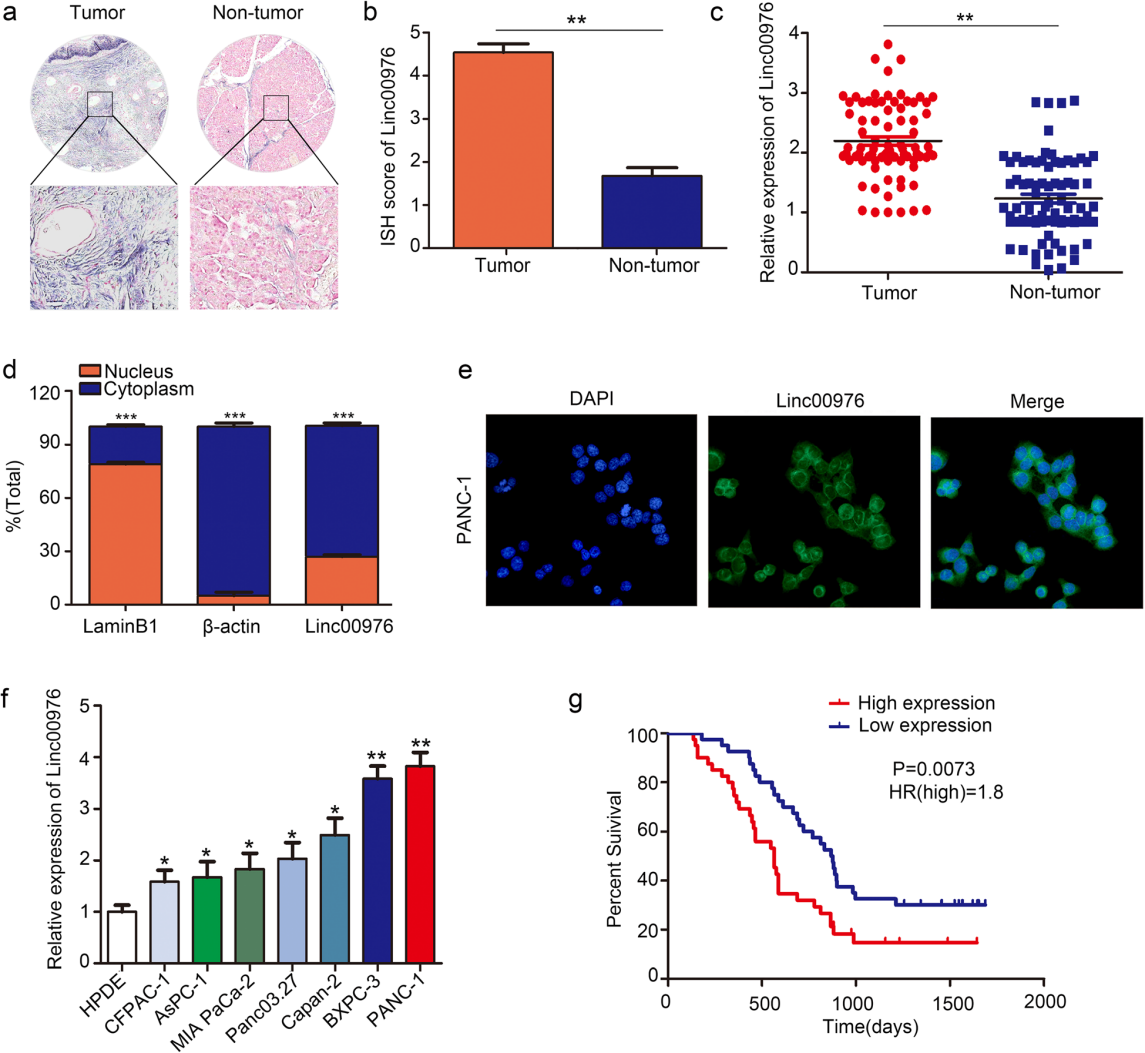
Linc00976 in pancreatic cancer tissue, cell lines, and the subcellular localization of expression. **a** and **b** ISH analysis of the expression level of Linc00976 in pancreatic cancer and normal pancreatic tissue. **c** qRT-PCR analysis of relative expression level of Linc00976 in PC tissue and adjacent normal tissues. **d** qRT-PCR analysis of RNA obtained from cell nuclei and cytoplasm of PANC-1 cells. **e** RNA-FISH localization of Linc00976 in PANC-1 cells. The data are shown as means ± S.D. of three independent assays. **f** qRT-PCR analysis of relative expression levels of Linc00976 in pancreatic cancer cell lines and HPDE cells. **g** Kaplan-Meier curve showing survival in PC patients divided by Linc00976 expression. Patients were divided into high (red) and low expression group (blue) according to the median of Linc00976 expression. (*P < 0.05, ** *P* < 0.01, *** *P* < 0.001)

We examined the express level of Linc00976 in 90 cases of human PC tissues and adjacent noncancerous tissues, the results of ISH and RT-qPCR revealed that the expression of Linc00976 was higher in tumor tissues than that in normal tissues (Fig. 1a-c). The nuclear extracted RNA fractionation and FISH assay demonstrated that Linc00976 was mainly located in the cytoplasm (Fig. 1d, e). The RT-qPCR was used to determine the expression of Linc00976 in PC cell lines (CFPAC-1, AsPC-1, Mia-Paca2, Panc03.27, BxPC-3, PANC-1) was significantly elevated compared with that in human pancreatic epithelium immortalized cells (HPDE) (Fig. 1f). According to the correlation of Linc00976 expression and the PC clinical pathology features (Table 2), We found that Linc00976 expression was significantly correlated with tumor size (*P* < 0.01), lymph node metastasis (*P* < 0.01), Perineural invasion (*P* < 0.01), vascular invasion (*P* < 0.05) and distant metastasis ability (*P* < 0.05). Further, Kaplan–Meier analysis illustrated that the Linc00976 expression highly patients had poorer prognosis than those Linc00976 expression lowly patients (*P* = 0.0073, HR = 1.8) (Fig. 1g).

**Table 2:** Association of Linc00976 expression with clinicopathological features from PC patients

Linc00976 Expression					
Features	*n*	Low	High	X^2^	*P*-value
All cases	126	63	63		
Age				0.519	0.471
<60	72	38	34		
≥60	54	25	29		
Gender				0.296	0.586
Man	75	39	36		
Female	51	24	27		
Tumor size (cm)				9.184	0.002
<2	61	39	22		
≥2	65	24	41		
Lymph node metastasis				9.651	<0.002
Negative	49	33	16		
Positive	77	30	47		
TNM stage				7.943	<0.005
I and II	43	29	14		
III and IV	83	34	49		
Distant metastasis				4.715	0.03
Negative	52	32	20		
Positive	74	31	43		
Perineural invasion				7.15	0.007
Negative	61	38	23		
Positive	65	25	40		
Blood vessel invasion				5.387	0.02
Negative	59	36	23		
Positive	67	27	40		

### Linc00976 promotes the proliferation of pancreatic cancer cells in vitro

**Fig. 2:**
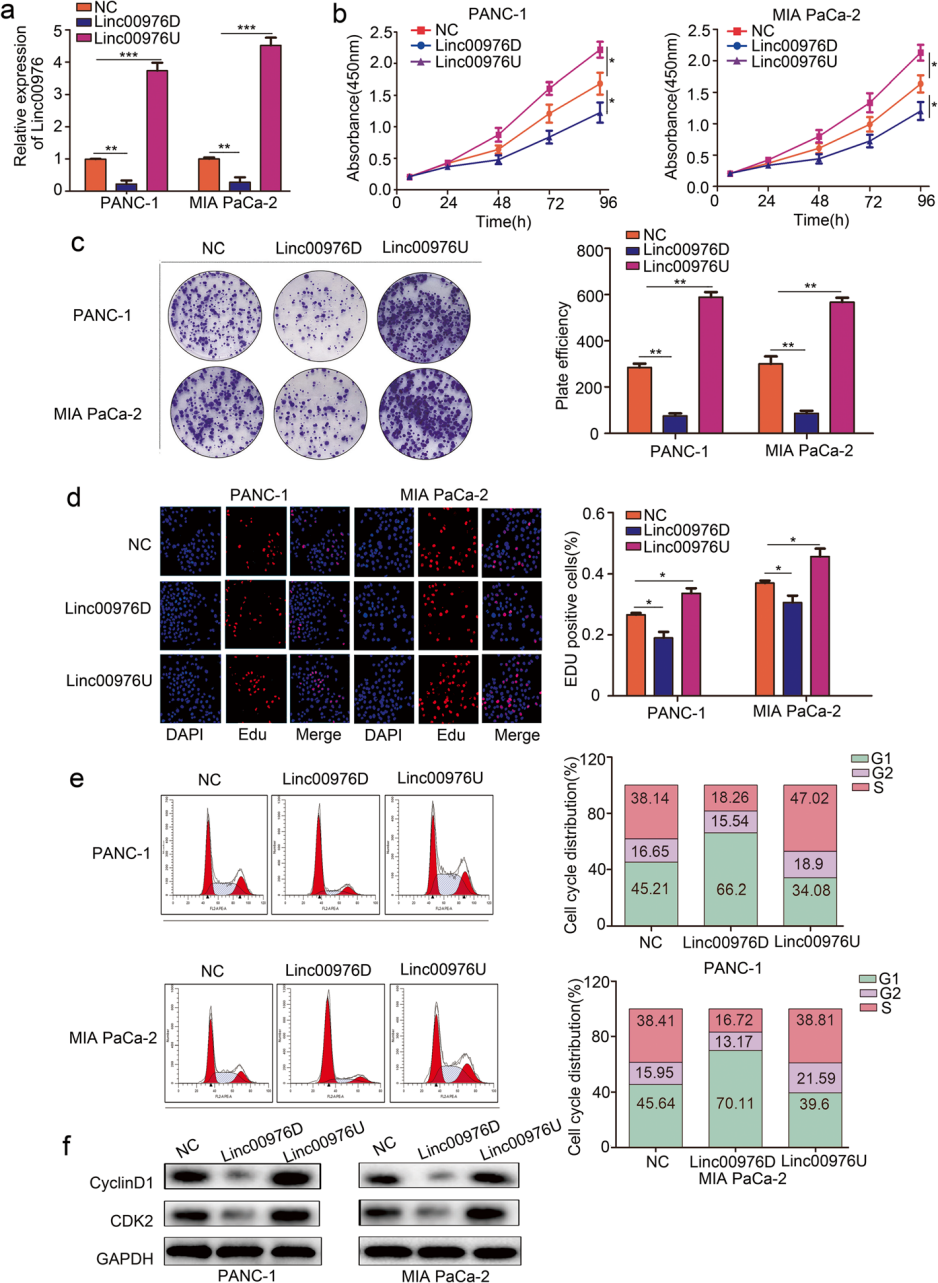
Linc00976 promotes cell proliferation of PC cells in vitro. **a** qRT-PCR analysis showing Linc00967 expression after transfection of Linc00976-knockdown (Linc00976D), Linc00976-overexpression (Linc00976U) and negative control (NC) in PANC-1 and MIA PaCa-2 cells infected by lentiviral infection. **b** CCK-8 assay comparing cell proliferation of Linc00976D, Linc00976U and NC group in PANC-1 and MIA PaCa-2 cells. **c** Colony formation experiment analysis the cell colony growth of Linc00976D, Linc00976U and NC group in PANC-1 and MIA PaCa-2 cells. **d** EDU assay analysis the proliferation capacity of Linc00976D, Linc00976U and NC group in PANC-1 and MIA PaCa-2 cells. **e** Effects of Linc00976 overexpression and Linc00976 knockdown on cell cycle progression in PANC-1 and MIA PaCa-2 cells. **f** Western-blot analysis the cell cycle associated protein Cyclin D1 and CDK2 expression of Linc00976D, Linc00976U and NC group in PANC-1 and MIA PaCa-2 cells. The data are shown as means ± S.D. of three independent assays (*P < 0.05, ** *P* < 0.01, *** *P* < 0.001)

Given that Linc00976 is overexpressed in PC tissues and cell lines, we further investigated the role of Linc00976 in proliferation. Stable Linc00976 overexpress (Linc00976U) and Linc00976 knockdown (Linc00976D) PANC-1 and Mia-paca2 stable cell lines were constructed with the lentiviral transfection (Fig. 2a). The proliferation assay eluted that Linc00976D PC cells resulted in significantly growth slower and formed fewer colonies compared with negative control (NC) cells, and that Linc00976U PC cells grew faster than NC cells (Fig. 2b–d). The cell cycle assay indicated that, Linc00976 knockdown shown a cycle block of PC cells in G0/G1 phase, with the S phase decreasing in the PC cells. In contrast, Linc00976 overexpression resulted in a significantly decrease in PC cells in G0/G1 phase, while the the proportion of S-phase exerting a significant rise (Fig. 2e). The western-blot assay was used to detected the G0/G1 phase regulating protein cyclin D1 and CDK2, the result revealed that cyclin D1 and CDK2 downregulated in Linc00976 knockdown PC cells, and upregulated in Linc00976 overexpressed PC cells (Fig. 2f).

### Linc00976 promotes the migration and invasion of pancreatic cancer cells in vitro

**Fig. 3:**
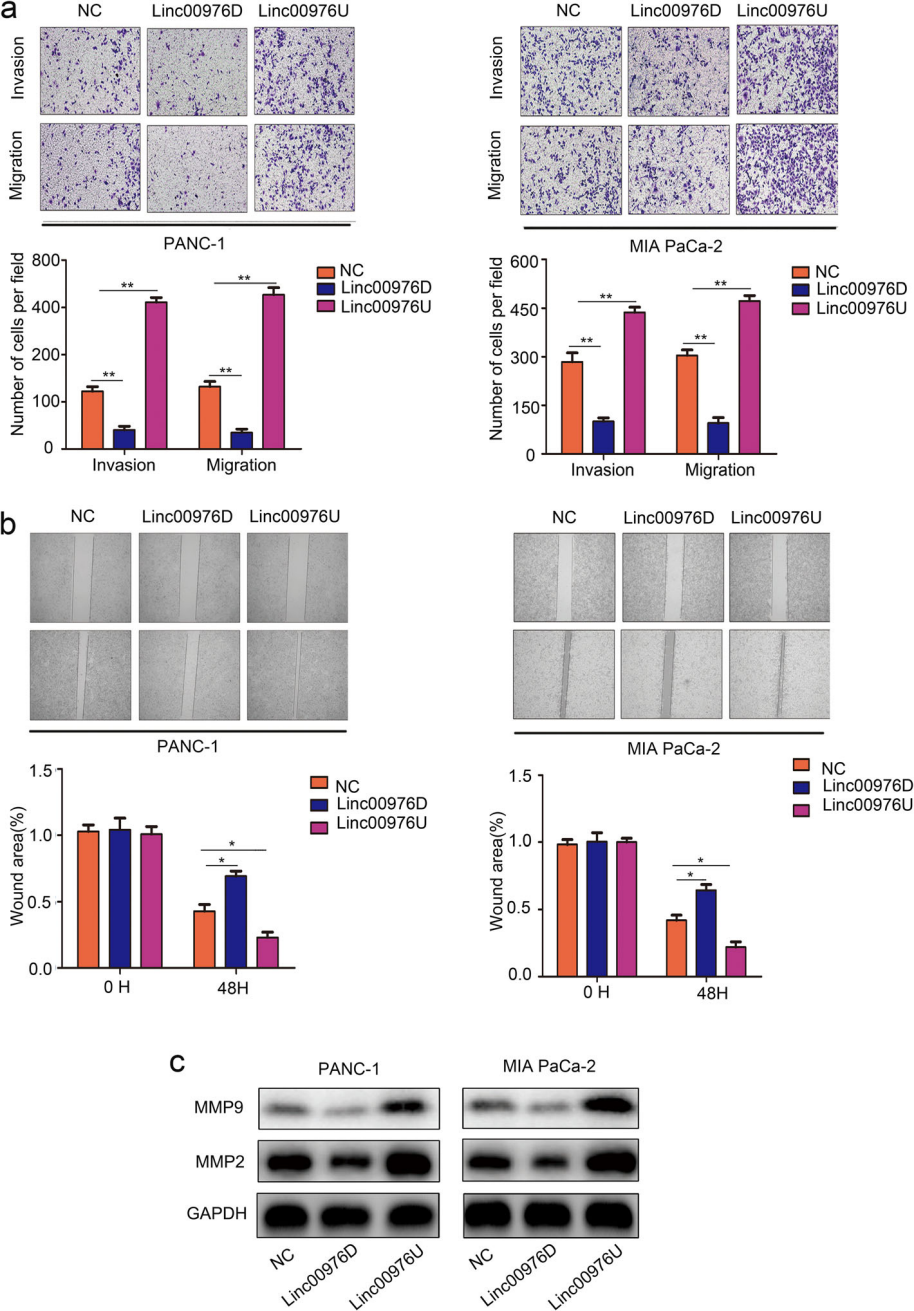
Linc00976 promotes the migration and invasion of PC cells in vitro. **a** Transwell assays analysis the migration and invasion ability of Linc00976D, Linc00976U and NC group in PANC-1 and MIA PaCa-2 cells. **b** Wound healing tests analysis the migration ability of Linc00976D, Linc00976U and NC group in PANC-1 and MIA PaCa-2 cells. **c** Western blot assays were used to measure MMP2 and MMP9 expression in PANC-1 and MIA PaCa-2 cells cells transfected with NC, Linc00976D, Linc00976U lentivirus

Further to explore the role of Linc00976 in PC cells migration and invasion ability. Transwell assays illustrated that Linc00976-knockdown cells showed lower migratory and invasive activities compared with control cells, while Linc00976-overexpressing cells showed higher migratory and invasive activities (Fig. 3a). The wound-healing assay also demonstrated that Linc00976-knockdown group exerted a lower migrasive ability compared to the NC group, whereas Linc00976 overexpression promoted migration (Fig. 3b). In the western blot assay, Linc00976 knockdown decreased MMP2 and MMP9, which promote cell migration and invasion (Fig. 3c).

### Linc00976 promotes PC cell proliferation, migration and invasion in vivo

**Fig. 4:**
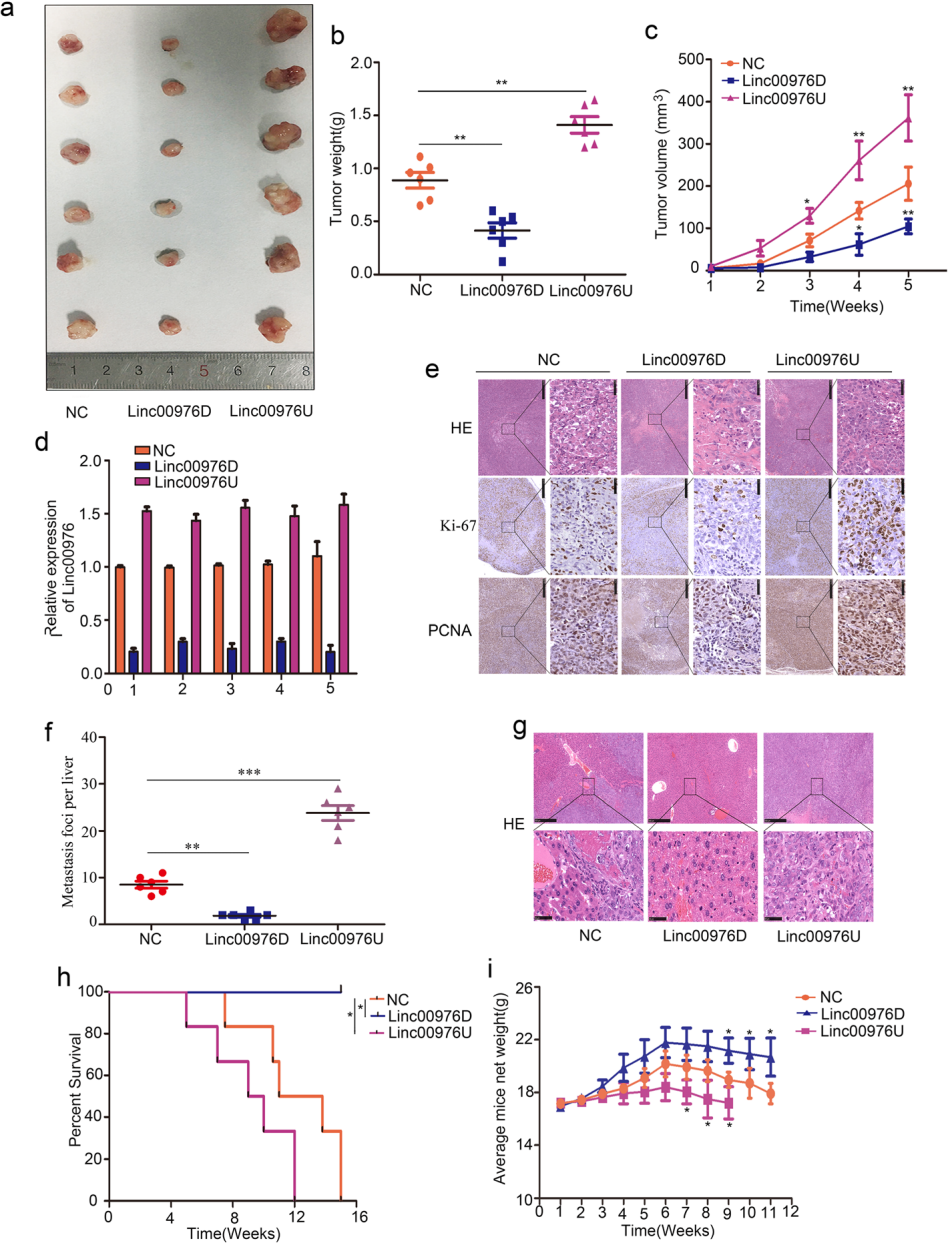
Linc00976 promotes the proliferation, invasion and metastasis of PC cells in vivo. **a** Typical image of nude mouse tumors (*n* = 6), **b** subcutaneous tumor weight, and **c** subcutaneous tumor volume, and **d** qRT-PCR assays showing the expression of Linc00976 in transplanted tumors. **e** Typical IHC staining images showing Ki-67 and PCNA expression in transplanted tumors under different experimental conditions. **f** the metastasis foci of per liver in liver metastasis model (*n* = 6). **g** Typical IHC staining of HE images showing metastasis loci in liver in indicated groups. **h** Kaplan-Meier survival curves for each experimental group (NC, Linc0097U and Linc00976D), for each group, *n* = 6. **i** Body weight changes for PANC-1 Linc00976U, Linc00976D and NC mice provided as mean ± SD, where each data point represents a different mouse

The xenograft tumor model and spleen capsule injected liver metastasis model were used to illustrate the different groups PC cells proliferation and metastasis ability in vivo. Six representative tumors from each group at 30th day were photographed (Fig. 4a). Linc00976 knockdown inhibited the xenograft tumor growth compared to the NC group, whereas Linc00976 overexpression had the opposite result (Fig. 4b, c). RT-qPCR illustrated that Linc00976 expression is downregulated in the xenograft tumors of Linc00976D group, and upregulated in the Linc00976U group (Fig. 4d). Immunohistochemistry (IHC) staining showed Ki-67 and PCNA positive rate was higher in the xenograft tumors of the Linc00976U when compared to the NC group, and lower in those of the Linc00976D group (Fig. 4e). The intrasplenically injected induce hepatic metastasis model showed fewer metastatic nodules in the liver tissues of nude mice injected with Linc00976D PANC-1 cells in the abdominal cavity, while Linc00976 overexpression caused more metastatic nodules (Fig. 4f). Hematoxylin and eosin (H&E) staining of liver tissue section showed that Linc00976D reduced the number of liver metastase loci compared with NC group. In contrast, Linc00976D increased the number of liver micrometastasis (Fig. 4g). According to the survival analyses, the Linc00976D group had the longer survival time, and Linc00976U had the shorter survival time (Fig. 4h). The weight lost more rapid in Linc00976D group, while slower in the Linc00976U group (Fig. 4i).

### Linc00976 promotes proliferation, migration and invasion by targeting OTUD7B in PC

**Fig. 5:**
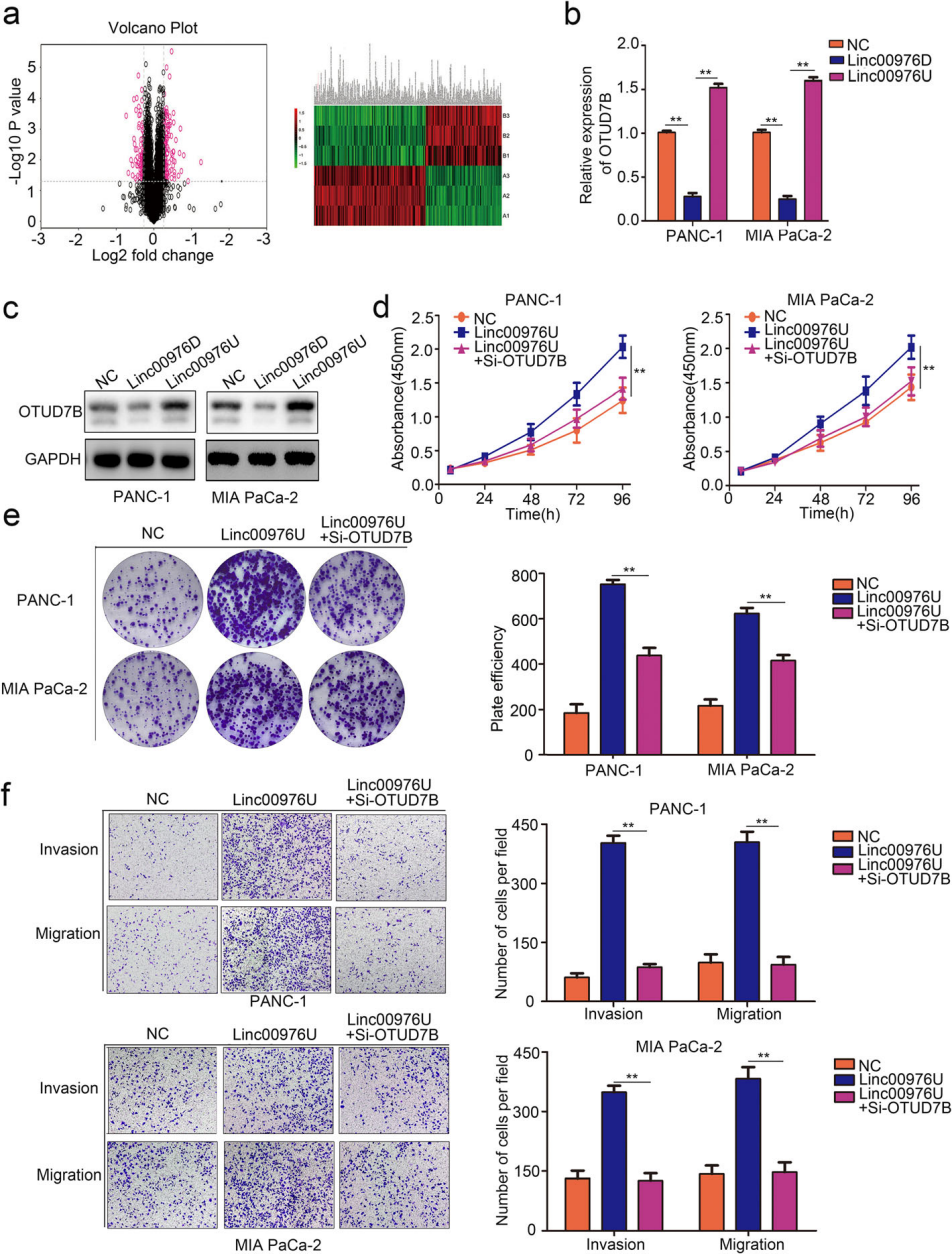
Linc00976 promotes proliferation, migration and invasion by targeting OTUD7B in pancreatic cancer. **a** iTRAQ analysis the express different proteins of Linc00976D, and NC group in PANC-1 cell. **b** and **c** RT-qPCR and Western blot assays analysis the mRNA and protein levels of OTUD7B in Linc00976D, Linc00976U and NC group PC cells. **d** and **e** CCK8 and colony formation assay showed that OTUD7B downregulated could partly suppress the proliferation which were promoted by Linc00976. **f** Transwell assay showed that OTUD7B downregulated could partly suppress the migration and invasion which were promoted by Linc00976

The changes to related proteins after Linc00976 downregulation were detected by iso-heavy isotope multilabel relative quantitative proteomics using iTRAQ analysis, the result showed that the expression of 136 proteins were increased, together with 93 proteins decreased while Linc00976 knockdown (Fig. 5a). Considering the proteins quantification analysis result (Additional file 3: Table S1), we hypothesis that Linc00976 may bring to bear the biological function via the downstream gene OTUD7B. Linc00976 knockdown significantly inhibited the mRNA and protein levels of OTUD7B, while Linc00976U upregulated expression (Fig. 5b–c). To verify whether Linc00976 exerted its role by upregulating OTUD7B in PC cells, we downregulated OTUD7B in Linc00976 overexpressed PC cells contributed for rescue experiments. RT-qPCR and western blot analysis showed that OTUD7B expression is upregulated in the linc00976U PC cells, while OTUD7B expression is partly decreased with OTUD7B silenced (Additional file 1: Figure S1). CCK8 and colony formation assay eluted that inhibition of OTUD7B attenuated the proliferation increased caused by Linc00976 overexpression (Fig. 5d, e). Transwell assays showed that OTUD7B knockdown could partly restrain the effect of Linc00976U on the migrative and invasive abilities of PC cells (Fig. 5f).

### OTUD7B deubiquitinated EGFR and facilitates downstream pathway MAPK

**Fig. 6:**
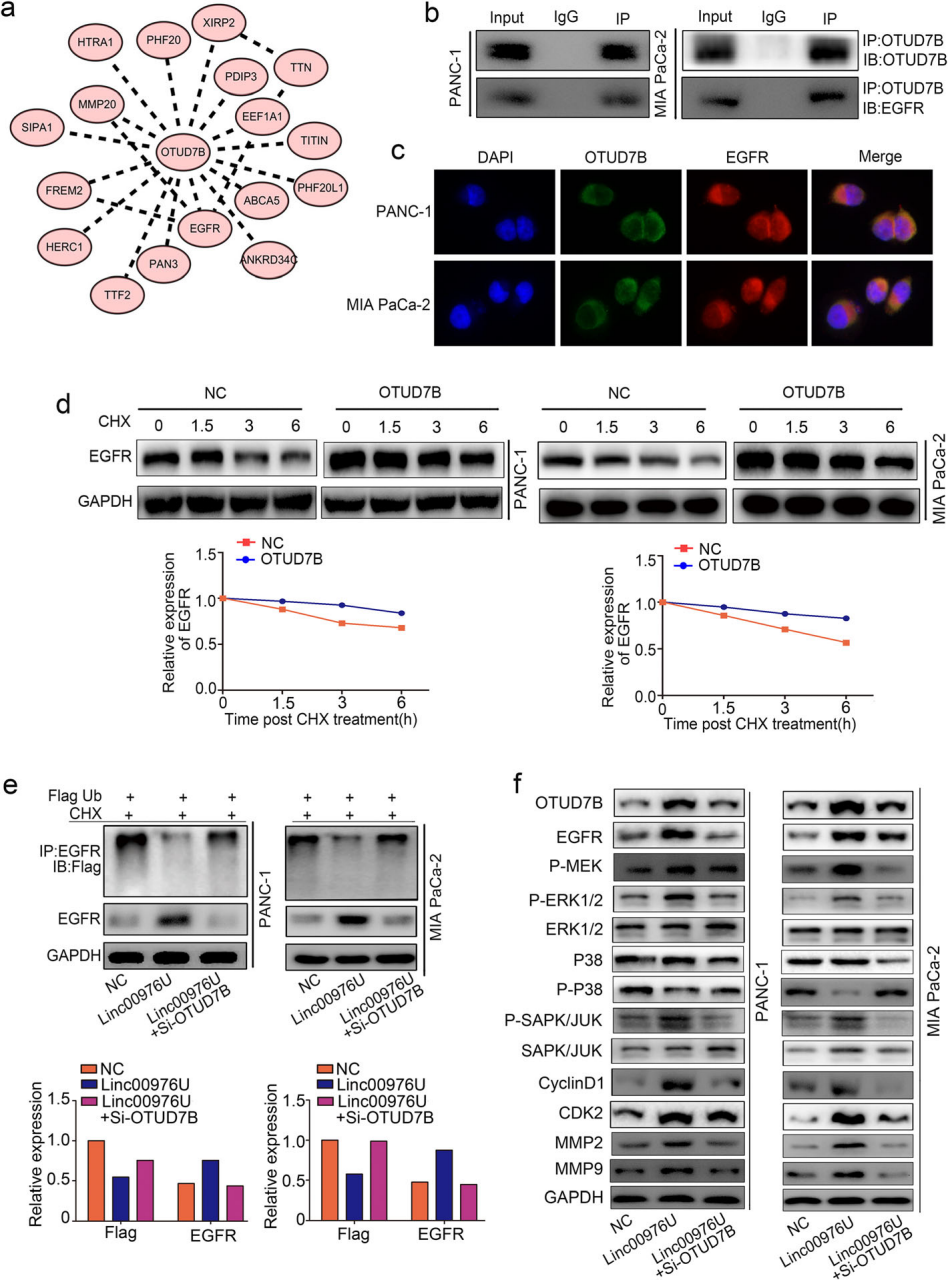
Linc00976 promotes OTUD7B-mediated EGFR deubiquitination and MAPK pathway activation. **a** Cytoscape showed the OTUD7B interaction proteins base on the Immunoprecipitation-MS (Mass Spectrometry). **b** and **c** Co-immunoprecipitation and immunofluorescent staining analysis the interaction between OTUD7B and EGFR. **d** The protein degradation assay analysis the stability of EGFR in OTUD7B overexpress NC group cells over the indicated time. **e** The ubiquitination assay showed that the ubiquitination level of EGFR in OTUD7B overexpressed and downregulated PC cells. **f** Western-blot indicated the expression and activity in EGFR downstream pathway MAPK in OTUD7B overexpressed and downregulated PC cells

Next we investigated the mechanism of host gene OTUD7B in Linc00976 mediating PC tumorigenesis. We used immunoprecipitation with mass spectrometry analysis to determine proteins interacting with OTUD7B (Additional file 4: Table S2). Then we captured the downstream genes interaction through iTRAQ results base on cytoscape (Fig. 6a). As the previous reports, OTUD7B regulated deubiquitination and endocytosis of EGFR associated with breast cancer proliferation, migration and malignancy [18]. Subsequently, the interaction between OTUD7B and EGFR was confirmed by co-immunoprecipitation and immunofluorescent staining (Fig. 6b, c). The protein degradation assay was used to detect the stability of EGFR revealed that EGFR were considerably more stable in OTUD7B overexpress cells than in NC group cells over the course of 6 h (Fig. 6d). The ubiquitination assay showed that ectopic expression of OTUD7B could increase the amount of poly-ubiquitinated EGFR, and the EGFR ubiquitination level was partly restrained in OTUD7B downregulated PC cells (Fig. 6e). Furthermore, the western-blot was used to eluted the EGFR downstream signal pathway MAPK, the results demonstrated the Linc00976 significantly activated the MAPK pathway, with the phosphorylated ERK, MEK, P38, JUK, and target gene Cyclin D1, CDK2, MMP2, MMP9 expression upregulating, while inhibited in OTUD7B downregulated PC cells (Fig. 6f).

### Linc00976-miR-137/OTUD7B axis on the EGFR downstream MAPK pathway

**Fig. 7:**
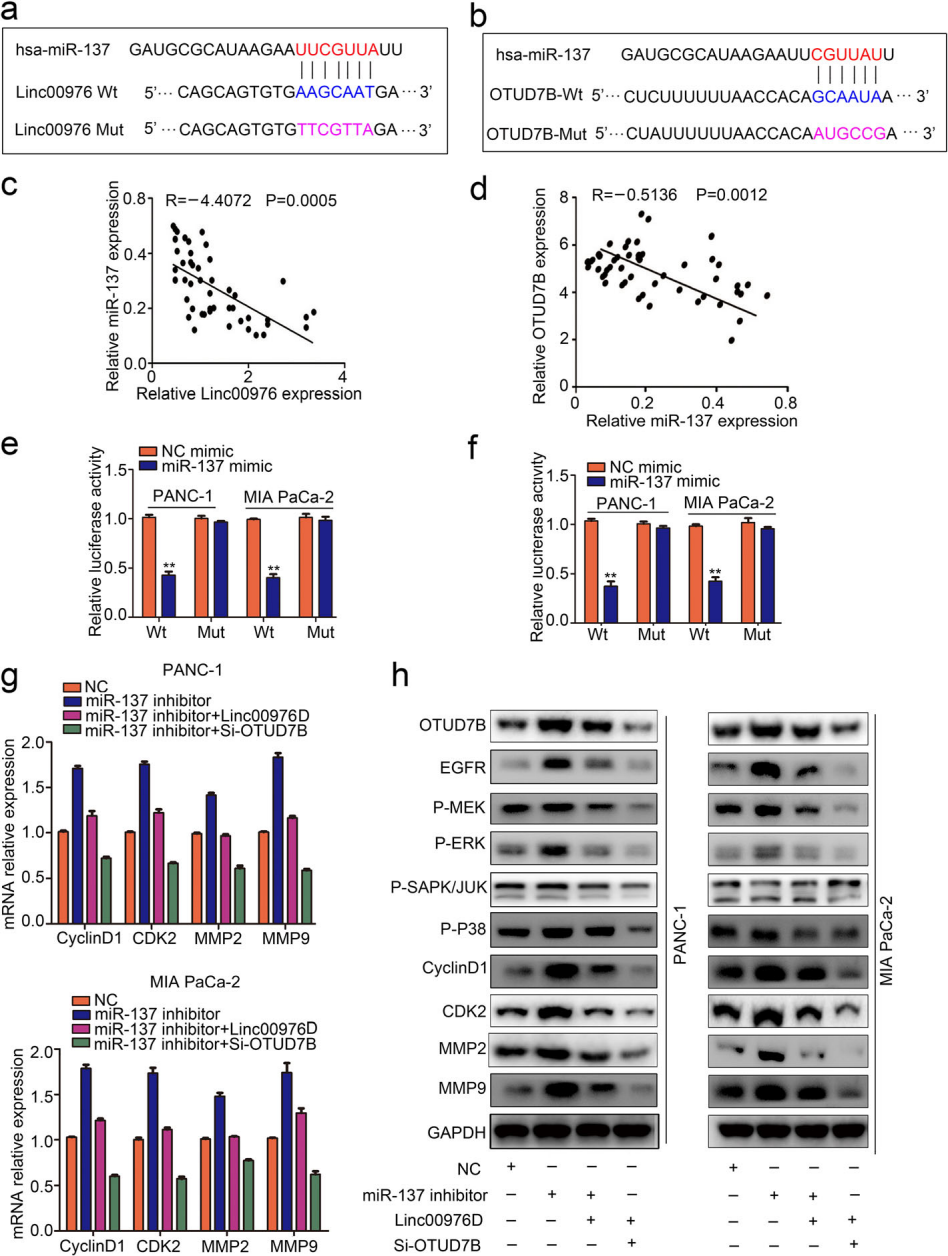
Linc00976-miR-137/OTUD7B axis on the EGFR downstream MAPK pathway. **a** and **b** The predicted potential binding site of miR-137 with wild-type Linc00976 (Linc00976-WT) and OTUD7B(OTUD7B-WT), and the designation of mutant of Linc00976 (Linc00976-MUT) and OTUD7B(OTUD7B-MUT) according to the seed region of the miR-137 binding site. **c** and **d** The Spearman rank correlation showed that negatively correlation with miR-137and Linc00976, OTUD7B. **e** and **f** Luciferase reporter assay analysis that the interaction with miR-137 and Linc00976, OTUD7B. **g** qRT-PCR showed that miR-137 overexpress and OTUD7B downregulated could partly restrained the mRNA express level of MAPK target genes (CyclinD1, CDK2, MMP2, MMP9) in Linc00976U PC cells. **h** Western-blot assay analysis of Linc00976/miR-137/OTUD7B axis on the EGFR downstream pathway MAPK activity

We thus aimed to determine whether Linc00976 affect post-transcriptional regulation with miRNA and function as miRNA sponges to impair the binding of miRNAs to OTUD7B. Base on the miRNA target sites by bioinformatics prediction, the 3’UTR of Linc00976 and OTUD7B bears potential miR-137 binding sites (Fig. 7a, b). Our previous research had revealed that miR-137 is downregulated and inhibits the growth, invasion, stemness in PC cell [19]. RT-qPCR assay analysis results showed that miR-137 expression was negatively correlated with Linc00976 and OTUD7B expression (Fig. 7c, d). miR-137 upregulated could impair the luciferase activity increased after transfected with the Linc00976-WT and OTUD7B-WT vector, while had no obvious impact on that of cells transfected with Linc00976-MUT or OTUD7B-MUT vector (Fig. 7e, f). These data revealed that Linc00976/miR-137/OTUD7B constructed a ceRNA regulation correlation. qRT-PCR illustrated that miR-137 downregulated promotes the mRNA express level of MAPK target genes (CyclinD1, CDK2, MMP2, MMP9), Linc00976 and OTUD7B downregulating could reverse the effect in Linc00976U PC cells (Fig. 7g). Subsequently, the western-blot assay was used to evaluate Linc00976/miR-137/OTUD7B axis on the EGFR downstream pathway MAPK, the results indicated that the miR-137 downregulated significantly activated the MAPK pathway, with the phosphorylated ERK, MEK, P38, JUK, and target gene Cyclin D1, CDK2, MMP2, MMP9 expression upregulating, while the effect was reversed by Linc00976 and OTUD7B downregulation (Fig. 7h). Furthermore, the CCK8, clone formation and transwell assay revealed Linc00976/miR-137/OTUD7B axis promotes the proliferation and metastasis in PC cells, while the linc00976 and OTUD7B downregulating could restrained the effect (Additional file 2: Figure S2a-b).

## Discussion

Recently, lncRNAs have been known as the focus in the field of cancer research involved in the biological and pathological progress of proliferation, metastasis, tumor metabolism and chemoresistance [20]. In order to identify whether Linc00976 was abnormally expressed in PC and its effect on prognosis, ISH and qRT-PCR was used to verify the Linc00976 express level in pancreatic adjacent/tumor tissues and normal/cancer cell lines, and the over survival analysis was used to evaluate prognosis risk. Function studies revealed that linc00976 knockdown significantly suppressed cell proliferation, cell cycle progression, migration and invasion in vivo and in vitro. These findings illustrated that linc00976 might act as an oncogene and play an important role in the progression of PC.

Furthermore, we used iTRAQ analysis to determine the host gene of linc00976. Function studies revealed that OTUD7B acts as an oncogene promoting proliferation and metastasis in PC cells. OTUD7B is a member of the OTU family of deubiquitinases, which is associated with the tumor rapid progress and deterioration. The main function of ubiquitin proteases in the ubiquitin proteasome system is to remove ubiquitin protein substrates by chemically cleaving either the linkage between ubiquitin molecules in the chain, or between ubiquitin molecules and the substrate. The ubiquitin molecules released from the substrates are involved in the regulation of proteins in the cell, resulting in a feedback loop. In addition, we found that linc00976 overexpress promoted proliferation and metastasis, while the effect was reversed by OTUD7B downregulated. These findings indicated that OTUD7B was the function host gene of linc00976 mediating PC tumorigenesis.

Research has repeatedly shown that OTUD7B is overexpressed and hold the vital role in in various types of cancers progress including hepatocellular carcinoma [21, 22] lung adenocarcinoma [23, 24], Breast [25], squamous cell carcinoma [26]. In addition, OTUD7B as a crucial immune regulator of NF-KB pathway involving Zap70 and TRAF3 deubiquitination and stabilization [27, 28]. However, the mechanism deubiquitinating enzyme OTUD7B promoted PC progress is not clear. Based on the results of Mass spectrometry of Immunoprecipitation proteins interacting with OTUD7B, EGFR may be the substrate in the OTD7B mediated PC tumorigenesis. As the previous report that OTUD7B can act on EGFR deubiquitination and endocytosis to affect the activation of the EGFR signaling pathway. The protein stability and ubiquitination assay eluted OTUD7B may inhibit EGFR ubiquitination, promote the EGFR protein stability. Accumulating evidence indicates that MAPK pathway contributes to the kinds of tumor physiological and biochemical progress, which is the substrate of activated EGFR. Therefore, we hypothesized that OTUD7B may deubiquitinate EGFR and activate downstream pathway MAPK. In addition to defining the regulation ship between OTUD7B and the proliferation capacity of PC, we found that the EGFR/MAPK pathway is involved in this regulation. Preliminary evidence on the role of activated MAPK pathway as mediators of EGFR promoted cancer tumorigenesis. Therefore, we found that Linc00976 promoted proliferation and invasion via MAPK pathway, which is activated by OTUD7B mediated EGFR deubiquitination and stability.

However, the mechanism of linc00976 regulating OTUD7B expression was still not clear. Currently, numerous lncRNAs that serve as endogenous miRNA sponges have been shown to be involved in the initiation and development of PC [29]. Long non-coding RNA XIST exerts oncogenic functions in pancreatic cancer via miR-34a-5p [30].

The lncRNA UCA1 promotes ovarian cancer cell chemoresistance by sponging miR-143 to activate FOSL2 Signaling Pathway [31]. FISH assay and nuclear RNA extraction was shown that linc00976 mainly locate in the cytoplasm. According to the prediction of bioinformatics software in this study, there were combination sites between linc00976 and miR-137.Our previous research revealed that miR-137 was a tumor pressor in pancreatic cancer growth, invasion and stemness [19]. We also found in this study, there were combination sites between Linc00976 and miR-137. Dual-luciferase reporter gene assay confirmed the regulation of Linc00976 on miR-137 and proved Linc00976 could directly bind to miR-137. At the same time, bioinformatics softwares also predicted that there were combination sites between miR-137 and OTUD7B. Dual-luciferase reporter gene assay proved miR-137 could directly bind to OTUD7B. And we found miR-137 express level was negative correlative with Linc00976 and OTUD7B expression in PC, which was consistent with the ceRNA regulation network in Linc00976/ miR-137/ OTUD7B. Further mechanistic studies indicated that Linc00976 exerts oncogene function by competitively interacting with miR-137 and acting as a microRNA sponge, thus inhibiting miR-137 and upregulating OTUD7B expression.

## Conclusion

**Fig. 8:**
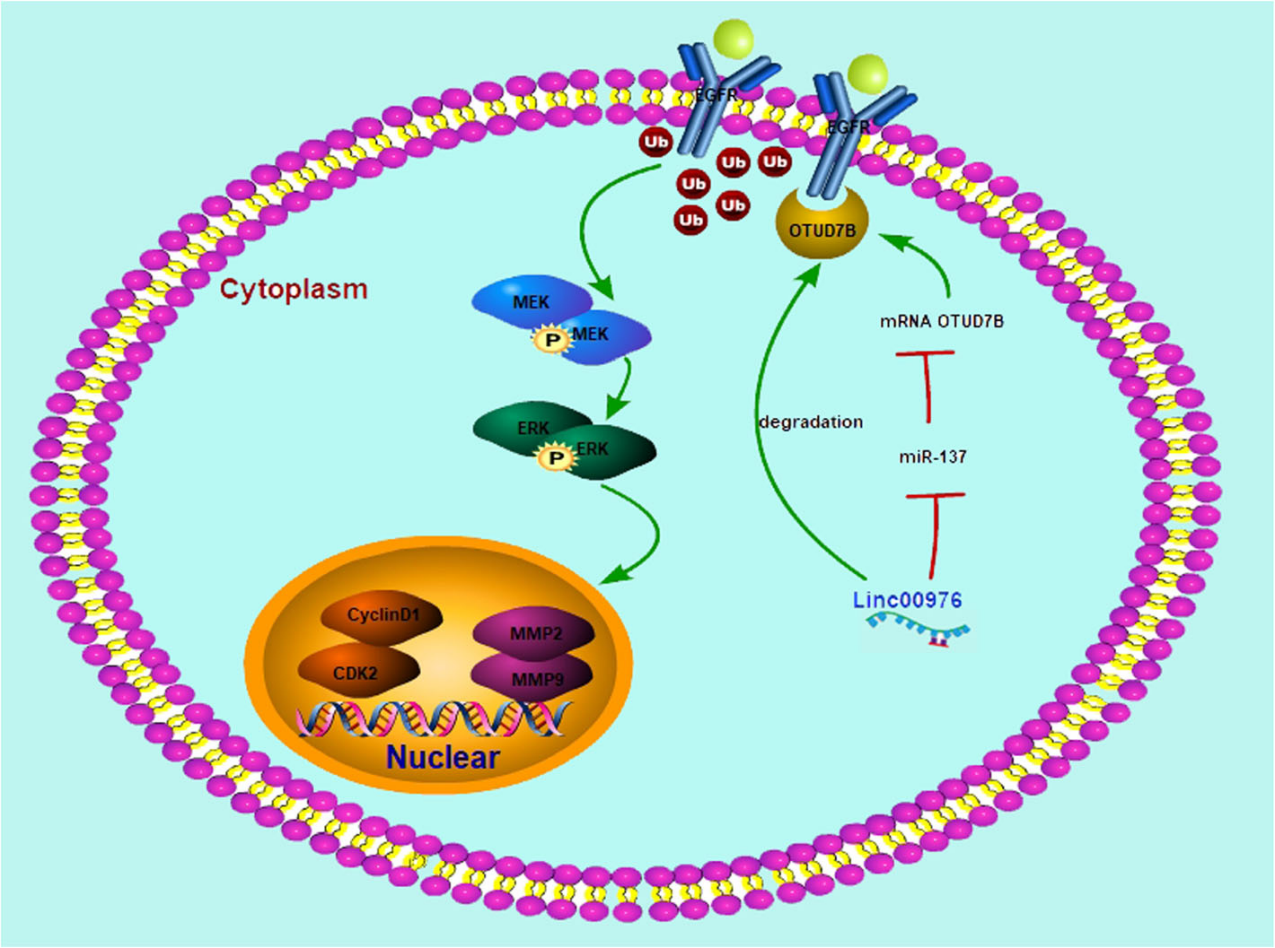
Long noncoding RNA 00976 promotes pancreatic cancer progression through OTUD7B by sponging miR-137 involving EGFR/MAPK pathway

As shown in Fig. 8, the present study demonstrates that linc00976 enhances the proliferation and metastasis ability of PC cells by sponging miR-137 to upregulate OTUD7B expression. Ultimately, OTUD7B mediates EGFR deubiquitination and stability and MAPK signaling pathway activation, suggesting that linc00976/miR-137/OTUD7B/EGFR axis may act as a feasible therapeutic target for PC.

## Additional files

Additional file 1: **Figure S1.** (a, b) RT-qPCR and western blot analysis the OTUD7B expression in linc00976U and linc00976 with OTUD7B silenced. (TIF 922 kb)

Additional file 2: **Figure S2.** (a) CCK8, clone formation and transwell assay elvaluated miR-137 overexpress and OTUD7B downregulated could partly restrained the proliferation promoted by Linc00976U. (b) Transwell assay illustrated that miR-137 overexpress and OTUD7B downregulated could partly restrained the migration and invasion promoted by Linc00976U. (TIF 18132 kb)

Additional file 3: **Table S1.** Different proteins in Linc00976 knockdown and NC PANC-1 based on the iTRAQ results. (XLSX 1041 kb)

Additional file 4: **Table S2.** Mass spectrometry of Immunoprecipitation proteins interacting with OTUD7B. (XLSX 935 kb)

## Data Availability

The datasets used and/or analyzed during the current study are available from the corresponding author on reasonable request.

## Abbreviations

CCK-8: Cell counting kit
lncRNA: Long non-coding RNA
miR: microRNA
OTUD7B: Ovarian tumor proteases 7
PC: Pancreatic cancer
qRT-PCR: Real-time quantitative reverse transcription PCR
