# Supplementary figures and images for: Long noncoding RNA 00976 promotes pancreatic cancer progression through OTUD7B by sponging miR-137 involving EGFR/MAPK pathway
Source: J Exp Clin Cancer Res. 2019 Nov 20;38:470. doi: 10.1186/s13046-019-1388-4 (PMC6868788; doi:10.1186/s13046-019-1388-4)

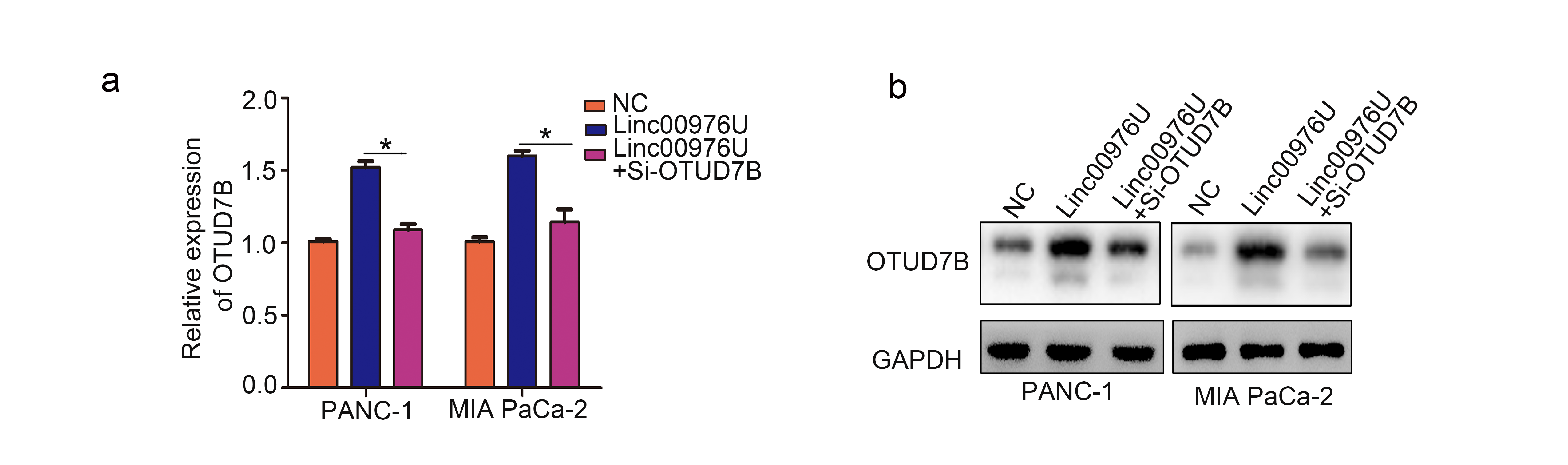

Supplement: Supplementary file 1 — Figure S1. (a, b) RT-qPCR and western blot analysis the OTUD7B expression in linc00976U and linc00976 with OTUD7B silenced. (TIF 922 kb) [file 13046_2019_1388_MOESM1_ESM.tif]

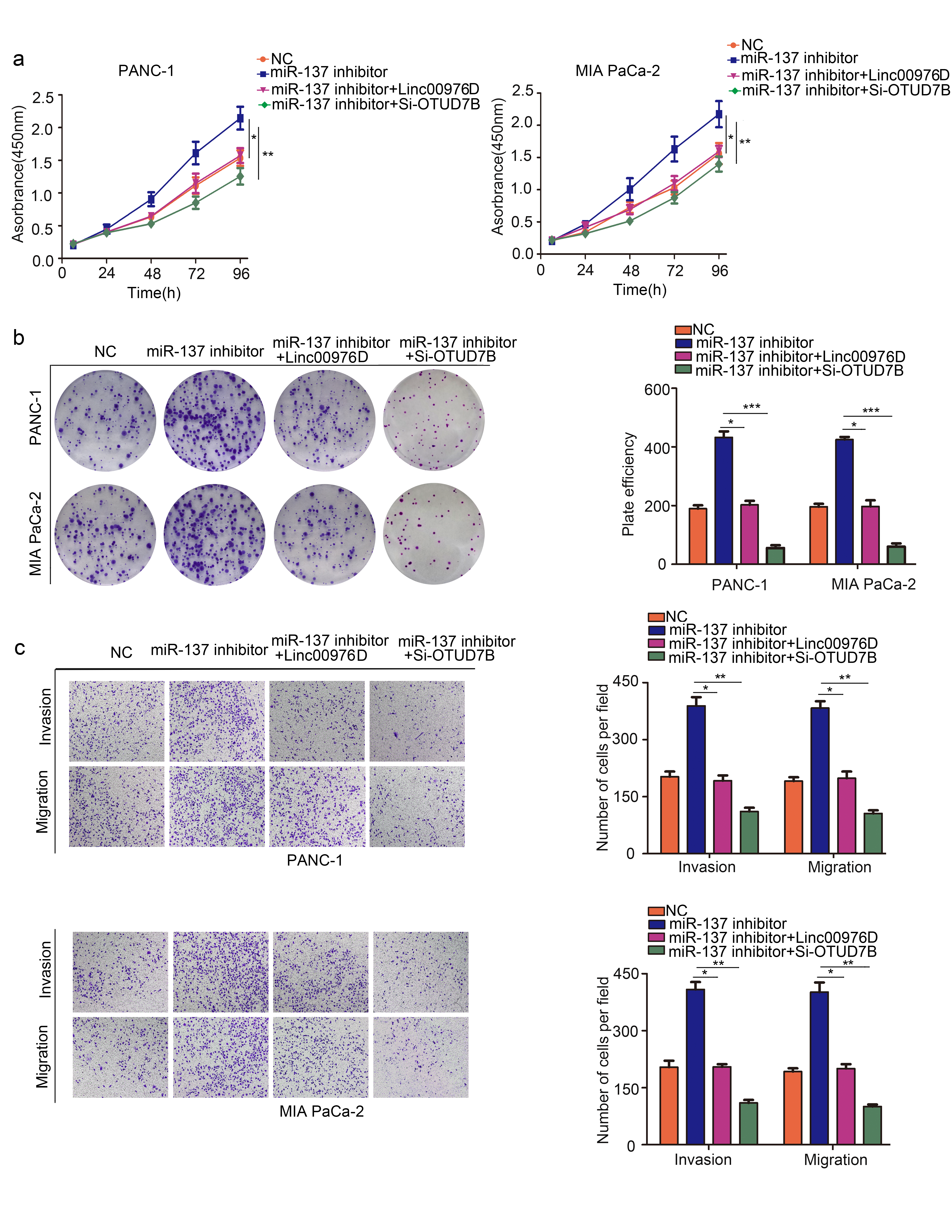

Supplement: Supplementary file 2 — Figure S2. (a) CCK8, clone formation and transwell assay elvaluated miR-137 overexpress and OTUD7B downregulated could partly restrained the proliferation promoted by Linc00976U. (b) Transwell assay illustrated that miR-137 overexpress and OTUD7B downregulated could partly restrained the migration and invasion promoted by Linc00976U. (TIF 18132 kb) [file 13046_2019_1388_MOESM2_ESM.tif]
